# Ixekizumab improves spinal pain, function, fatigue, stiffness, and sleep in radiographic axial Spondyloarthritis: COAST-V/W 52-week results

**DOI:** 10.1186/s41927-021-00205-3

**Published:** 2021-09-20

**Authors:** Atul A. Deodhar, Philip J. Mease, Proton Rahman, Victoria Navarro-Compán, Vibeke Strand, Theresa Hunter, Rebecca Bolce, Luis Leon, Steve Lauzon, Helena Marzo-Ortega

**Affiliations:** 1 Division of Arthritis and Rheumatic Diseases, Oregon Health & Science University, 3181 Sam Jackson Park Rd, Portland, OR 97239 USA; 2 Swedish Medical Center/ Providence St. Joseph Health and University of Washington, Seattle, WA USA; 3 Memorial University of Newfoundland, St. John’s, NL Canada; 4 Department of Rheumatology, Hospital Universitario La Paz, IdiPAZ, Madrid, Spain; 5 Division of Immunology/Rheumatology, Stanford University School of Medicine, Palo Alto, CA USA; 6 Eli Lilly and Company, Indianapolis, IN USA; 7 National Institute for Health Research (NIHR) Leeds Biomedical Research Centre, Leeds Teaching Hospitals Trust and LIRMM, University of Leeds, Leeds, WY UK

**Keywords:** ASAS, PROs, Spinal pain, Stiffness, Fatigue, Radiographic axial spondyloarthritis, Ixekizumab

## Abstract

**Background:**

This analysis assessed improvements in patients with radiographic axial spondyloarthritis (r-axSpA) treated with ixekizumab in the Assessment of Spondyloarthritis International Society (ASAS) treatment response domains and additional patient-reported outcomes at 1 year of treatment.

**Methods:**

COAST-V and COAST-W were 52-week, phase 3, randomized controlled trials evaluating the efficacy and safety of ixekizumab in biologic disease-modifying antirheumatic drug (bDMARD)-naïve and tumor necrosis factor inhibitor (TNFi)-experienced patients with radiographic spondyloarthritis, respectively. Patients were treated with 80-mg ixekizumab either every 2 weeks or every 4 weeks. Patient-reported outcomes included Patient Global Disease Activity, Spinal Pain, stiffness as measured by Bath Ankylosing Spondylitis Disease Activity Index (BASDAI) Questions 5 and 6, function as measured by the Bath Ankylosing Spondylitis Functional Index, fatigue as measured by the Fatigue Numeric Rating Scale and BASDAI question 1, Spinal Pain at Night, and sleep quality as measured by the Jenkins Sleep Evaluation Questionnaire. Mixed-effects models for repeated measures were used to analyze changes from baseline in patient-reported outcomes from weeks 1 to 16, and descriptive statistics were reported from weeks 20 to 52. Analysis of covariance with Scheffé’s method was used for the ASAS response association analyses.

**Results:**

This study assessed 341 bDMARD-naïve and 316 TNFi-experienced patients in the placebo-controlled blinded treatment dosing period (weeks 1–16) as well as 329 bDMARD-naïve and 281 TNFi-experienced patients in the dose double-blind extended treatment period (weeks 20–52). bDMARD-naïve or TNFi-experienced patients treated with ixekizumab every 2 weeks and every 4 weeks reported improvements in patient global disease activity, spinal pain, function, stiffness, fatigue, spinal pain at night, and sleep quality through week 52. Greater correlations with improvements in all response domains were seen when comparing ASAS40 responders to ASAS20 non-responders (*p* < 0.001), with up to 10.5-fold greater improvements observed in ASAS40 responses compared with ASAS20 non-responders. Function and fatigue demonstrated the highest values.

**Conclusions:**

Ixekizumab-treated bDMARD-naïve and TNFi-experienced patients with radiographic axial spondyloarthritis achieving ASAS40 reported sustained and consistent improvement in all ASAS response domains and other patient-reported outcomes though week 52, with spinal pain, function, and stiffness as major drivers of the response.

**Trial registration:**

NCT02696785 and NCT02696798, March 2, 2016.

**Supplementary Information:**

The online version contains supplementary material available at 10.1186/s41927-021-00205-3.

## Background

Radiographic axial spondyloarthritis (r-axSpA) is a chronic inflammatory condition that affects the axial skeleton and is also referred to as ankylosing spondylitis (AS). Up to 0.5% of adults worldwide are reported to have the disease [1–3]. R-axSpA is differentiated from non-radiographic axial spondylarthritis by the presence of definite sacroiliitis that can be seen on plain radiographs [4]. Patients with r-axSpA experience negative changes to their health-related quality of life with common symptoms including spinal pain, stiffness, and sleep disruption, which can improve with treatment [5].

Ixekizumab is a high-affinity monoclonal antibody that selectively targets interleukin (IL)-17A, a proinflammatory cytokine. In the context of r-axSpA, ixekizumab has demonstrated efficacy in patients who are naïve to biologic disease modifying antirheumatic drugs (bDMARD) and in patients intolerant of or inadequate responders to tumor necrosis factor inhibitors (TNFi); ixekizumab treatment in these two patient populations were evaluated in the COAST-V and COAST-W trials, respectively [6, 7].

The Assessment of Spondyloarthritis International Society (ASAS) response criteria (with the domains patient global disease activity [PtGA], spinal pain, function, and stiffness), which include ASAS20, ASAS40, and ASAS partial remission, are commonly used to measure r-axSpA treatment efficacy in clinical research [8]. The COAST-V and COAST-W trials applied ASAS40 as the primary endpoint, a higher standard than ASAS20 [6, 7, 9, 10]. In the clinical setting, however, clinicians focus on the most significant individual patient-reported outcomes (PROs) including stiffness, fatigue, spinal pain at night, and sleep quality. At week 16, ixekizumab demonstrated significantly greater improvements (2.6 to 5.3-fold and 5.1 to 18.5-fold in TNFi-experienced and bDMARD-naïve patients, respectively) in fatigue, spinal pain at night, and sleep over placebo in patients with r-axSpA who achieved ASAS40 compared with ASAS20 non-responders [10]. Here, we evaluate the impact on ASAS response criteria domains of ixekizumab treatment in patients with r-axSpA through 52 weeks, as well as the impact on PROs such as fatigue, spinal pain at night, and sleep quality and their associations with achieving ASAS40.

## Methods

### Study design

COAST-V (NCT02696785) and COAST-W (NCT02696798) were phase 3, multicentre, randomised, double-blind, active (COAST-V only) and placebo-controlled, 52-week trials. The trial designs for COAST-V and COAST-W have been previously published [6, 7, 9]. Briefly, patients in both studies who were assigned to ixekizumab were randomised on a 1:1 ratio to receive a starting dose of either 80 mg or 160 mg. The efficacy and safety of ixekizumab was assessed for 80 mg every 2 weeks (Q2W) and every 4 weeks (Q4W) compared with placebo during a 16-week placebo-controlled blinded treatment dosing period, followed by a dose double-blind extended treatment period (up to 52 weeks). COAST-V contained an active-reference arm including patients receiving 40 mg of adalimumab Q2W up to week 16. The trial protocols were approved by the ethics review boards at each study site. The master ethics committee was Schulman Associates IRB, Cincinnati, OH, USA (IRB # 201506061 and IRB # 201506079 for COAST-V and COAST-W, respectively), and full listings of investigators and sites are available in previously published manuscript supplements [6, 7]. Both trials were conducted in accordance with the ethical principles of the Declaration of Helsinki. All patients gave written informed consent prior to the start of trial-related procedures.

### Patients

Inclusion criteria for COAST-V and COAST-W have been previously described [6, 7, 9]. Eligible adult patients fulfilled the ASAS criteria for axSpA, including radiographic evidence of sacroiliitis according to modified New York criteria associated with ≥1 SpA feature. In addition, patients had a Bath Ankylosing Spondylitis Disease Activity Index (BASDAI) ≥4 and total back pain score ≥ 4 on a numeric rating scale (NRS) and inadequate response or intolerance to non-steroidal anti-inflammation drug therapy. COAST-V included only patients with no prior or current use of bDMARDs. COAST-W included only patients with prior treatment with 1 or 2 TNFis.

### Treatment protocol

The dosing period regimens for COAST-V and COAST-W have been published [6, 7, 9, 10]. Briefly, 341 patients in COAST-V and 316 in COAST-W were randomized to receive subcutaneous injections of 80-mg ixekizumab Q2W or Q4W, 40-mg adalimumab Q2W (COAST-V only), or placebo Q2W for 16 weeks. In the dose double-blind extended treatment period (weeks 16 to 52), patients who originally received adalimumab or placebo were rerandomised 1:1 to ixekizumab Q2W or Q4W, while patients originally randomised to receive ixekizumab Q2W or Q4W continued those treatments.

### Assessments

The primary endpoint for both COAST-V and COAST-W was the proportion of patients achieving an ASAS40 response at week 16. Secondary efficacy endpoints included ASAS20 and ASAS partial remission, and changes from baseline in ASAS treatment response domains (PtGA, spinal pain, function, and stiffness), in addition to the clinically impactful symptoms of fatigue, spinal pain at night, and sleep quality.

Function was evaluated as the average score of Bath Ankylosing Spondylitis Functional Index (BASFI) responses [8]. Stiffness was evaluated as the average score from responses to BASDAI questions 5 and 6 (BASDAI Q5 and Q6) [8]. Fatigue was evaluated by the Fatigue NRS [11] and by BASDAI question 1 (BASDAI Q1) [8]. Each ASAS response domain was scored on a scale from 0 to 10. Sleep quality was assessed by the Jenkins Sleep Evaluation Questionnaire (JSEQ) and scored on a scale from 0 to 20, with higher scores indicating greater sleep disturbances [12]. Assessments were made at weeks 0, 1, 2, 4, 8, 12, and 16 during the placebo-controlled blinded treatment dosing period [10], and weeks 20, 24, 28, 32, 36, 44, and 52 during the dose double-blind extended treatment period [9], except for Fatigue NRS and JSEQ where assessments were made at week 8, 16, 36, and 52.

Week 52 data were pooled across treatment groups to assess the relationship between improvements in individual PROs and the achievement of different levels of ASAS response. These pooled data were then stratified by ASAS response level: ASAS20 non-responders, ASAS20 but not ASAS40 responders, and ASAS40 responders.

### Statistical analyses

Efficacy and health outcome analyses for the placebo-controlled blinded treatment (weeks 0 to 16) and the dose double-blind extended treatment period (weeks 16 to 52) were conducted on all patients randomized to treatment groups in COAST-V and COAST-W. Comparisons between treatment groups, and least square mean changes from baseline for the ASAS treatment response domains and other PROs up to week 16 were analyzed using a mixed-effects model for repeated measures, as described previously [10]. The mixed-effects model included treatment, geographic region, baseline CRP status, baseline value, visit, baseline value-by-visit, and treatment-by-visit interaction as fixed factors, with variance-covariance structure set to unstructured. Number of prior TNFi was an additional fixed factor for the COAST-W model. After week 16, changes from baseline were summarized as mean and standard deviation for the ASAS treatment response domains and other PROs.

Post hoc association analyses were performed using data from week 52 between ASAS response levels and changes from baseline in the four ASAS response domains (PtGA, spinal pain, function, stiffness) and the additional PROs fatigue, spinal pain at night, and sleep quality. From weeks 0 to 52, changes from baseline in these PROs were compared between the ASAS responder groups listed above using analysis of covariance (ANCOVA) models after adjusting for baseline values, age, and gender. Standard error and standard deviation were used as measures of dispersion for ANCOVA. Post hoc comparisons were conducted with Scheffé’s correction, which was based on multiple comparisons of outcome measures. Fold increases were determined by the following calculations: (ASAS40 responder/ASAS20 non-responder) -1 or (ASAS40 responder/ASAS20 but not ASAS40 responder) -1. Modified baseline observation carried forward (mBOCF) was used for imputation of missing data [13].

All analyses were conducted using SAS 9.4. Residual plots were generated via SAS to confirm normality of residuals.

## Results

### Baseline characteristics

This study assessed 341 bDMARD-naïve and 316 TNFi-experienced patients in the placebo-controlled blinded treatment dosing period (weeks 0 to 16) as well as 329 bDMARD-naïve and 281 TNFi-experienced patients in the dose double-blind extended treatment period (weeks 16 to 52). Baseline disease characteristics for the bDMARD-naïve and TNFi-experienced patients in the dose double-blind extended treatment period population are displayed in Table 1.

**Table 1:** Baseline disease characteristic for bDMARD- naïve and TNFi-experienced patients in COAST-V and COAST-W

	COAST-V: bDMARD-naïve (*N* = 329)		COAST-W: TNFi-experienced (*N* = 281)	
	Mean (range)	SD	Mean (range)	SD
Age, years	41.6 (19–78)	11.7	46.3 (18–76)	12.3
Duration of disease since diagnosis, years	7.7 (0–43.9)	8.4	11.4 (0.3–43.8)	9.2
ASAS treatment response domains				
PtGA	7.1 (1–10)	1.6	7.9 (0–10)	1.6
Spinal pain (BASDAI Q2)	7.3 (3–10)	1.5	8.2 (4–10)	1.3
Function (BASFI)	6.2 (0.8–10)	1.9	7.2 (0.7–10)	1.7
Stiffness (BASDAI Q5/Q6)	6.7 (1–10)	1.7	7.2 (2–10)	1.7
Other outcomes				
Fatigue (NRS)	6.8 (0–10)	1.7	7.3 (0–10)	1.7
Fatigue (BASDAI Q1)	7.1 (2–10)	1.6	7.6 (3–10)	1.4
Spinal pain at night	7.1 (2–10)	1.6	7.7 (0–10)	1.7
Sleep (JSEQ)	8.2 (0–20)	5.2	10.2 (0–20)	5.6
BASDAI	6.7 (2.8–10)	1.4	7.4 (3.4–10)	1.3

*ASAS* Assessment of Spondyloarthritis International Society, *BASDAI* Bath Ankylosing Spondylitis Disease Activity Index, *BASFI* Bath Ankylosing Spondylitis Functional Index, *bDMARD* biologic disease-modifying antirheumatic drugs, *JSEQ* Jenkins Sleep Evaluation Questionnaire, *N* number of patients in analysis population, *NRS* numeric rating scale, *PtGA* patient global disease activity, Q question, *SD* standard deviation, *TNFi* tumor necrosis factor inhibitor.

Ns and values are from the extended treatment period patient population. Data shown as mean (range) and SD

### Changes from baseline in ASAS treatment response domains

**Fig. 1:**
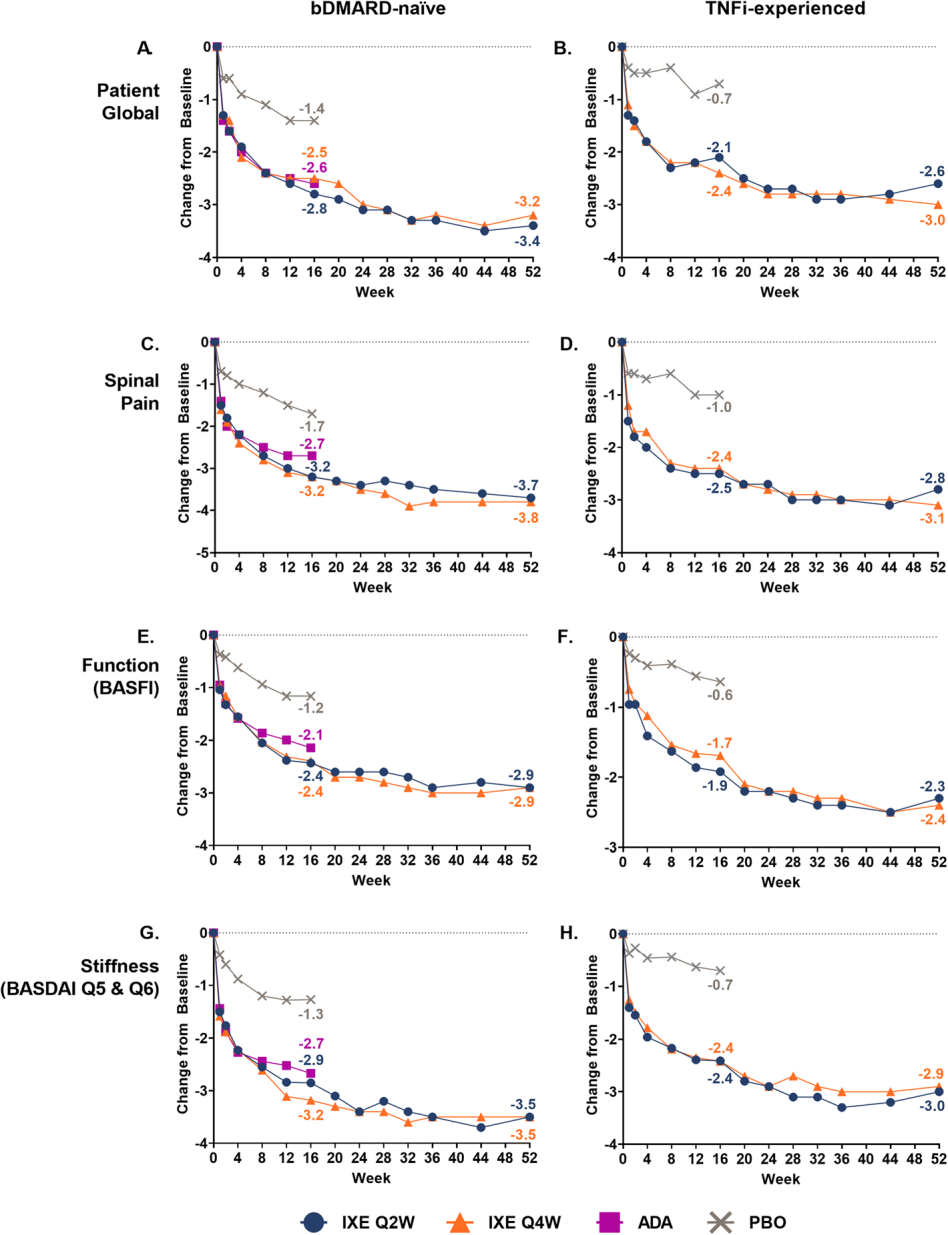
**Changes from baseline in the ASAS treatment response domains through 52 weeks of treatment**. LSM (weeks 1–16) and mean (weeks 20–52) changes from baseline in the ASAS treatments response domains (PtGA, spinal pain, function, and stiffness) for bDMARD-naïve (COAST-V) and TNFi-experienced (COAST-W) patients. The 16-week data have been published previously (10). At week 16, patients who originally received ADA or PBO were rerandomised 1:1 to ixekizumab Q2W or Q4W, while patients originally randomised to receive ixekizumab Q2W or Q4W continued treatment. ADA = adalimumab; ASAS = Assessment of Spondyloarthritis International Society; BASDAI = Bath Ankylosing Spondylitis Disease Activity Index; BASFI = Bath Ankylosing Spondylitis Functional Index; bDMARD = biologic disease-modifying antirheumatic drugs; IXE = ixekizumab; LSM = least squares mean; PBO = placebo; PtGA = patient global disease activity; Q = question; Q2W = every 2 weeks; Q4W = every 4 weeks; TNFi = tumor necrosis factor inhibitor

As previously reported, improvements were seen in the four ASAS treatment domains at week 16 in patients treated with ixekizumab compared with placebo [10]. Improvements in these measures were consistent over time in patients continuously treated with ixekizumab up to 52 weeks; these include PtGA, spinal pain, function, and stiffness (Fig. 1, Supplementary Table S1).

### Improvements in additional patient-reported outcomes

**Fig. 2:**
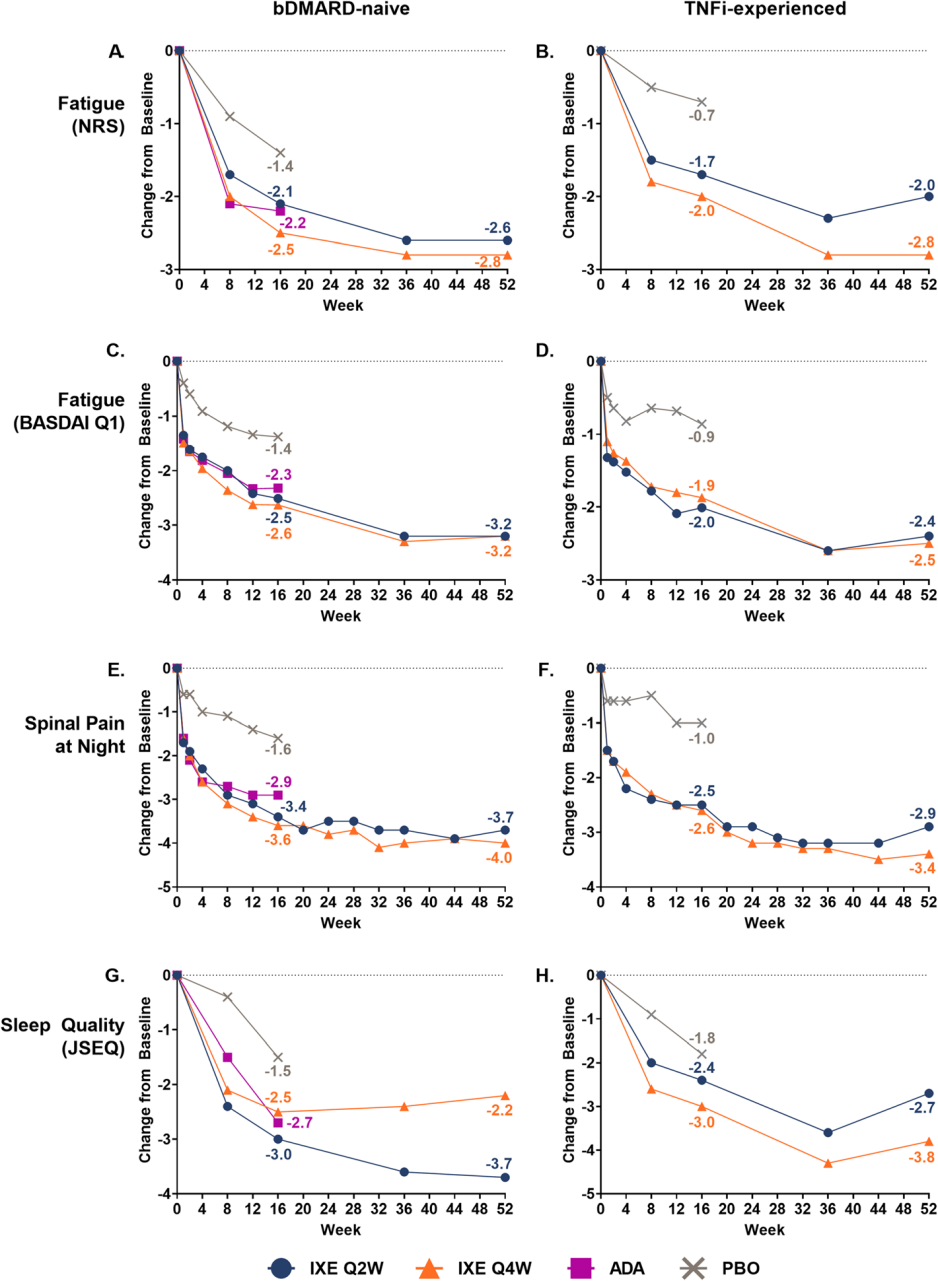
**Changes from baseline in other PROs through 52 weeks of treatment**. LSM (weeks 1–16) and mean (weeks 20–52) changes from baseline in other PROs for bDMARD-naïve (COAST-V) and TNFi-experienced (COAST-W) patients. The 16-week data have been published previously (10). At week 16, patients who originally received ADA or PBO were rerandomised 1:1 to ixekizumab Q2W or Q4W, while patients originally randomised to receive ixekizumab Q2W or Q4W continued those treatments. ADA = adalimumab; BASDAI = Bath Ankylosing Spondylitis Disease Activity Index; bDMARD = biologic disease-modifying antirheumatic drugs; IXE = ixekizumab; JSEQ = Jenkins Sleep Evaluation Questionnaire; LSM = least squares mean; NRS = numeric rating scale; PBO = placebo; PRO = patient-reported outcome; Q = question; Q2W = every 2 weeks; Q4W = every 4 weeks; TNFi = tumor necrosis factor inhibitor

Improvements were reported at 16 weeks for these outcomes in ixekizumab-treated patients compared with placebo [10], and these responses were sustained for patients continuously treated with ixekizumab up to 52 weeks in both studies (Fig. 2, Supplementary Table S1).

### Association of ASAS40 response with improvements in patient global disease activity, spinal pain, function, and stiffness

**Fig. 3:**
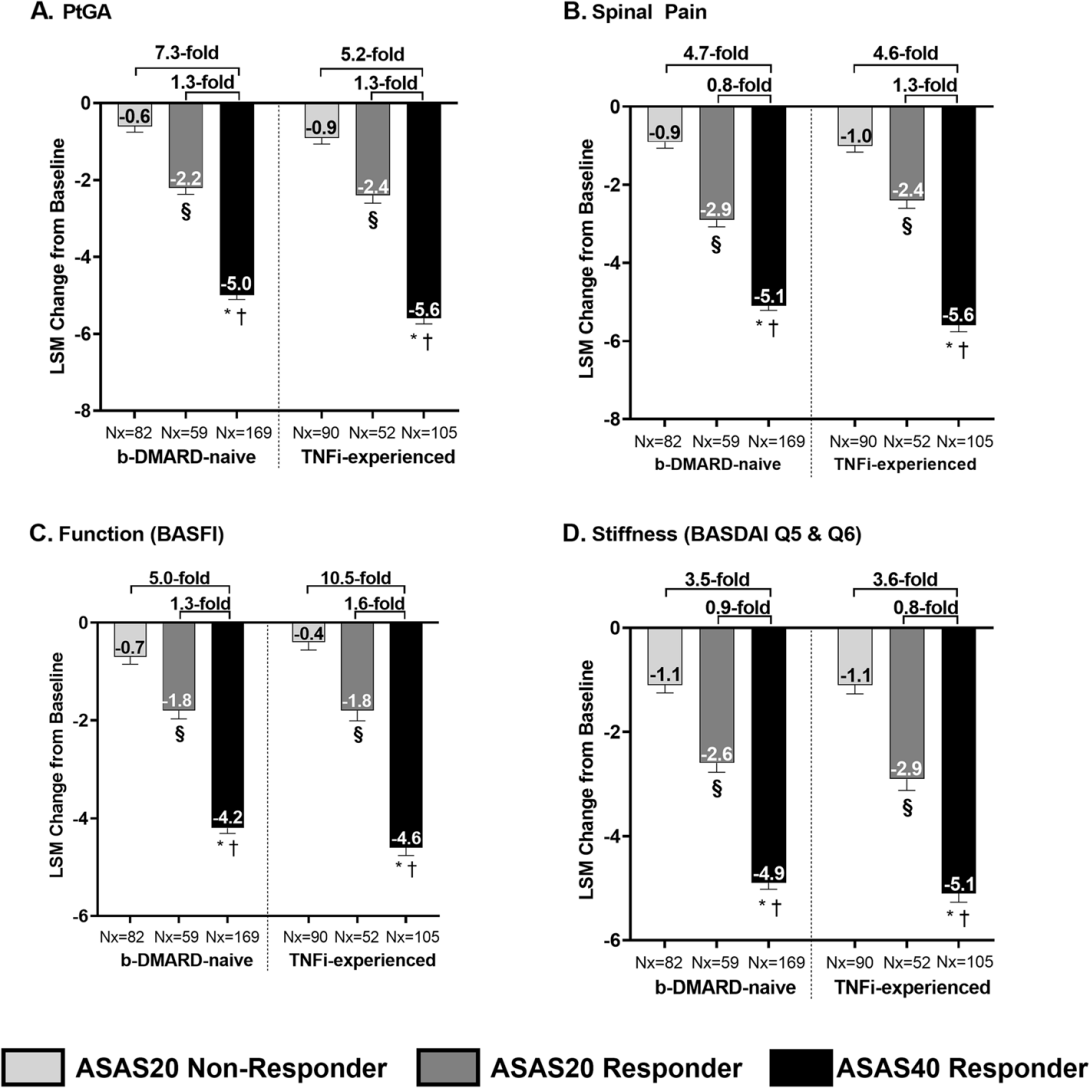
**Association between ASAS response and improvements in ASAS PROs for ixekizumab-treated patients after 52 weeks**. Q2W and Q4W ixekizumab-treated patients. § *p* < 0.001, achieved ASAS20 but not ASAS40 vs. ASAS20 not achieved; * *p* < 0.0001, ASAS40 achieved vs. ASAS20 not achieved; † p < 0.0001, ASAS40 achieved vs. achieved ASAS20 but not ASAS40. Results were compared using ANCOVA. Values are LSM improvements from baseline (SE). mBOCF was used for imputation of missing data. Fold difference = (ASAS40 responder/ASAS20 non-responder) -1, or (ASAS40 responder/ASAS20 but not ASAS40 responder) -1. ANCOVA = analysis of covariance; ASAS = Assessment of Spondyloarthritis International Society; BASDAI = Bath Ankylosing Spondylitis Disease Activity Index; BASFI = Bath Ankylosing Spondylitis Functional Index; bDMARD = biologic disease-modifying antirheumatic drugs; LSM = least squares mean; mBOCF = modified baseline observation carried forward; Nx = number of observations; PROs = patient-reported outcomes; PtGA = patient global disease activity; Q = question; Q2W = every 2 weeks; Q4W = every 4 weeks; SE = standard error; TNFi = tumor necrosis factor inhibitor

Changes from baseline in individual ASAS domains (PtGA, spinal pain, function, and stiffness) were compared at week 52 among three ASAS response groups in order to evaluate the relationship between individual PROs and the achievement of ASAS response for both bDMARD-naïve and TNFi-experienced ixekizumab (Q2W and Q4W)-treated patients (Fig. 3).

Greater improvements were reported for bDMARD-naïve patients who achieved ASAS40 when compared with ASAS20 non-responders. A 7.3-fold improvement (− 0.6 vs. -5.0, *p* < 0.0001) in PtGA, a 4.7-fold improvement (− 0.9 vs. -5.1, *p* < 0.0001) in spinal pain, a 5.0-fold improvement (− 0.7 vs. -4.2, *p* < 0.0001) in function, and a 3.5-fold improvement (− 1.1 vs. -4.9, *p* < 0.0001) in stiffness were observed. Greater improvements were also reported when TNFi-experienced patients who achieved ASAS40 were compared with ASAS20 non-responders. A 5.2-fold improvement (− 0.9 vs. -5.6, *p* < 0.0001) in PtGA, 4.6-fold improvement (− 1.0 vs. -5.6, *p* < 0.0001) in spinal pain, a 10.5-fold improvement (− 0.4 vs. -4.6, *p* < 0.0001) in function, and a 3.6-fold improvement (− 1.1 vs. -5.1, *p* < 0.0001) in stiffness was observed.

Improvement in several PROs was also calculated for both bDMARD-naïve and TNFi-experienced patients who achieved ASAS40 compared with those who achieved ASAS20, but not ASAS40. Significantly greater improvements (p < 0.0001) were also reported for the PROs of PtGA, spinal pain, function, and stiffness, for ixekizumab treated patients through 52 weeks of treatment, although more moderate (0.8-fold to 1.6-fold) than when compared with ASAS20 non-responders (Fig. 3).

### Association of ASAS responses with improvements in fatigue, spinal pain at night, and sleep quality

**Fig. 4:**
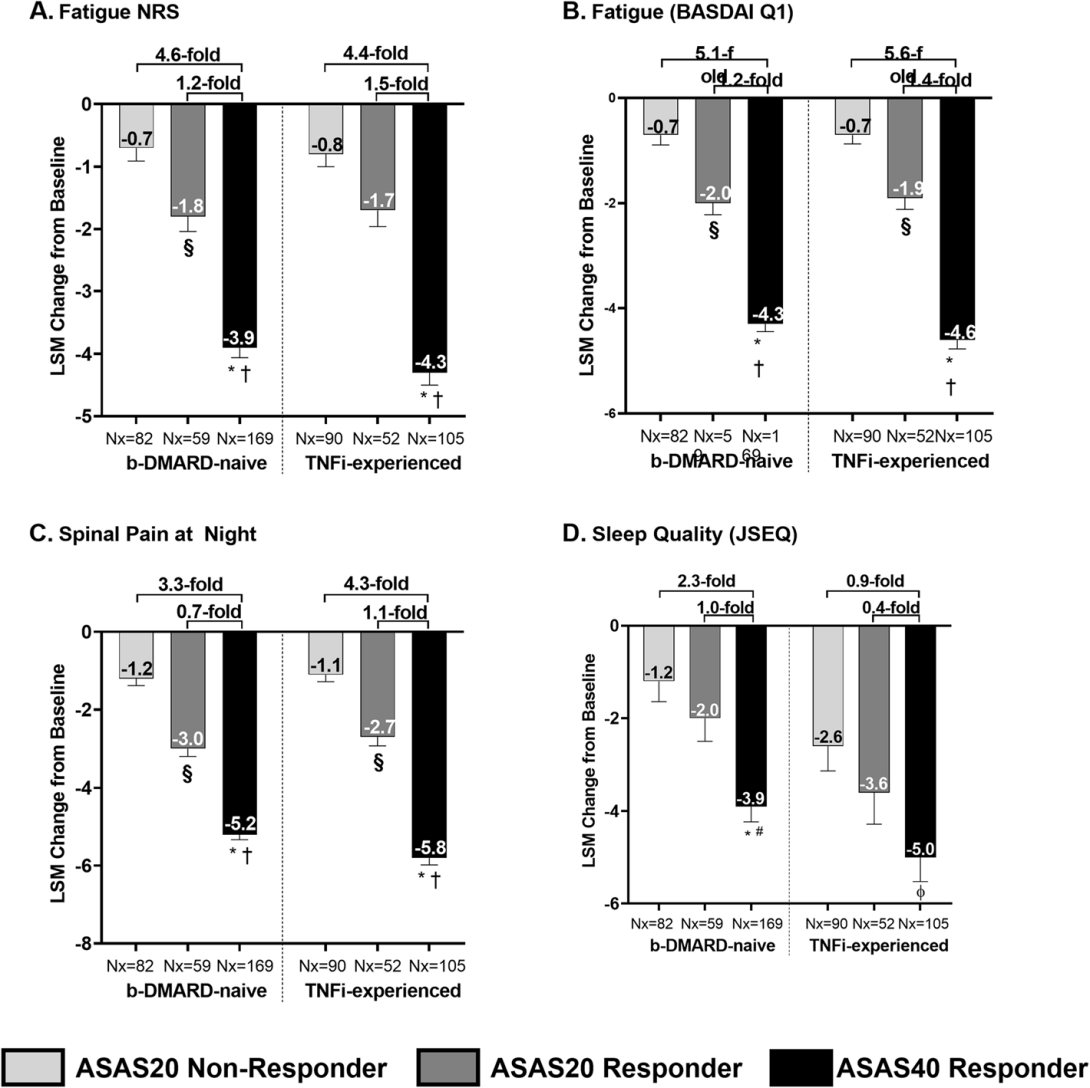
**Association between ASAS response and improvements in other PROs for ixekizumab-treated patients after 52 weeks**. Q2W and Q4W ixekizumab-treated patients. § *p* < 0.001, achieved ASAS20 but not ASAS40 vs. ASAS20 not achieved; Φ p < 0.001, * p < 0.0001, ASAS40 achieved vs. ASAS20 not achieved; # p < 0.001, † p < 0.0001, ASAS40 achieved vs. achieved ASAS20 but not ASAS40. Results were compared using ANCOVA. Values are LSM improvements from baseline (SE). mBOCF was used for imputation of missing data. Fold difference = (ASAS40 responder/ASAS20 non-responder) -1, or (ASAS40 responder/ASAS20 but not ASAS40 responder) -1. ANCOVA = analysis of covariance; ASAS = Assessment of Spondyloarthritis International Society; BASDAI = Bath Ankylosing Spondylitis Disease Activity Index; bDMARD = biologic disease-modifying antirheumatic drugs; JSEQ = Jenkins Sleep Evaluation Questionnaire; LSM = least squares mean; mBOCF = modified baseline observation carried forward; NRS = numeric rating scale; Nx = number of observations; PROs = patients reported outcomes; Q = question; Q2W = every 2 weeks; Q4W = every 4 weeks; SE = standard error; TNFi = tumor necrosis factor inhibitor

Continuous treatment with ixekizumab (Q2W and Q4W) demonstrated significant improvements from baseline in fatigue, spinal pain at night, and sleep quality for bDMARD-naïve patients who achieved ASAS40 when compared with ASAS20 non-responders. A 4.6-fold improvement (− 0.7 vs. -3.9, *p* < 0.0001) in fatigue NRS, a 5.1-fold improvement (− 0.7 vs. -4.3, *p* < 0.0001) in fatigue (BASDAI Q1), a 3.3-fold improvement (− 1.2 vs. -5.2, *p* < 0.0001) in spinal pain at night, and 2.3-fold improvement (− 1.2 vs. -3.9, *p* < 0.0001) in sleep quality were observed (Fig. 4).

Similar results were reported for the TNFi-experienced patients. When compared with ASAS20 non-responders, patients who achieved ASAS40 had significantly greater improvements from baseline. A 4.4-fold improvement (− 0.8 vs. -4.3, *p* < 0.0001) in fatigue NRS, a 5.6-fold improvement (− 0.7 vs. -4.6, *p* < 0.0001) in fatigue (BASDAI Q1), a 4.3-fold improvement in spinal pain at night (− 1.1 vs. -5.8, *p* < 0.0001), and a 0.9-fold improvement (− 2.6 vs.-5.0, *p* < 0.001) was observed for sleep quality (Fig. 4).

When patients who achieved ASAS40 were compared with those who achieved ASAS20 but not ASAS40, moderate but significantly greater improvements were seen (*p* < 0.001) in fatigue (NRS and BASDAI Q1), spinal pain at night, and sleep quality for bDMARD-naïve patients (Fig. 4).

It is important to note that similar results were observed when Q2W and Q4W ixekizumab-treated patients in both COAST-V and COAST-W trials were analyzed separately (Supplementary Figures S1-S4).

## Discussion

Clinical trials evaluating the efficacy of bDMARDs in r-axSpA have historically used ASAS20 as the primary endpoint [14–17]. The COAST-V and COAST-W trials assessed the efficacy of ixekizumab in r-axSpA using ASAS40 as the primary endpoint, a higher efficacy standard relative to ASAS20. Understanding the role of ixekizumab on the impact of achieving ASAS40 through improvements in PROs described herein will allow physicians to translate a higher ASAS achievement to the improvements in the signs and symptoms reported by their patients, directly affecting a patients’ overall function clinical outcome. In addition to the ASAS response domains and other PROs reported here, patients diagnosed with r-axSpA typically suffer from symptoms that negatively impact their quality of life including impaired work productivity. In this post hoc analysis, we report the durable impact of ixekizumab through 52 weeks of treatment on PROs and the individual association of these with ASAS responses. Responses for both dosing regimens (ixekizumab Q2W and ixekizumab Q4W) were similar for both bDMARD-naïve and TNFi-experienced patients across all endpoints. Numeric changes from baseline were observed in the ASAS response domains (PtGA, spinal pain, function, and stiffness) and other PROs (fatigue, spinal pain at night, and sleep quality), including overall disease activity through 52 weeks of treatment. Results were sustained and consistent for both the placebo-controlled blinded treatment (weeks 0 to 16) and the dose double-blind extended treatment period (weeks 16 to 52).

When ASAS40 responders were compared with ASAS20 non-responders, significant 3.5 to 10.5 -fold improvements were observed for the ASAS response domains (PtGA, spinal pain, function, and stiffness), and 2.3 to 5.6-fold improvements for other PROs (fatigue, spinal pain at night, and sleep quality). In this study, the major drivers responsible for achieving the ASAS40 response are PtGA (7.3-fold improvement), BASDAI fatigue (5.1-fold improvement), and function (5.0-fold improvement) for the bDMARD-naïve patients, and function (10.5-fold improvement) and BASDAI fatigue (5.6-fold improvement) for the TNFi-experienced patients. Patient-reported fatigue defined by BASDAI Q1 is a prevalent and major clinical feature of r-axSpA [18]. Therefore, the magnitude of improvement observed for fatigue in this study translates the clinical impact of achieving an ASAS40 response into PROs used in daily clinical practice, providing better management of r-axSpA.

A recent study investigated the effect of ixekizumab treatment in bDMARD-naïve and TNFi-experienced patients with r-axSpA on work productivity, including absenteeism, presenteeism, and overall work impairment through week 52 [19]. Patients from both trials reported greater improvements in work productivity from ixekizumab treatment compared with placebo at week 16, with improvements sustained and consistent through 52 weeks. Another study evaluated the effect of ixekizumab on functioning and health in r-axSpA patients from the COAST-V and COAST-W trials [20]. For both bDMARD-naïve and TNFi-experienced patients, ixekizumab treatment resulted in significant improvements in the Short Form Health Survey 36-item (SF-36), ASAS Health Index (ASAS HI), and the European Quality of Life-5 Dimensions-5 Levels EQ-5D-5L versus placebo at week 16 through week 52. The potential association between fatigue and work productivity is worth exploring as an additional analysis as fatigue and stiffness would have a direct effect on work productivity in patients suffering from r-axSpa. A recent study evaluating another IL-17 inhibitor, secukinumab, in the context of r-axSpA found that patients who achieved a remission status at week 156 in ASAS inactive disease, ASAS partial remission, and/or low BASDAI scores also reported improved PROs, which supports the relationship between r-axSpA remission and better health-related quality of life as determined by PROs [21]. Strengths of this study include the enrollment of a global patient population with an established diagnosis of r-axSpA from two dedicated studies to either bDMARD-naive and TNFi-experienced patients with relatively long disease duration who were followed for at least 1 year. In addition, this study evaluated a diverse array of PROs (ASAS components and other PROs) as well as their magnitude of benefit in the context of overall ASAS response by assessing fold changes at 52 weeks. A limitation of this study is that the association analyses on the correlation of ASAS treatment response and PROs was performed post hoc from COAST-V and COAST-W trial data. This analysis was also possibly limited by a relatively small number of ASAS20 responders.

## Conclusions

In conclusion, this post hoc analysis demonstrated the sustained and consistent effectiveness of ixekizumab in r-axSpA patients who achieved ASAS40 compared with patients who achieved ASAS20 or ASAS20 non-responders providing greater improvements in clinically relevant PROs including PtGA, spinal pain, spinal pain at night, function, stiffness, fatigue, and sleep quality through 1 year of treatment. Striving to reach an ASAS40 level of improvement will translate to better clinical disease management and improvement in common symptoms and patient quality of life.

## Supplementary Information

**Additional file 1: Table S1.** Changes from baseline in patient reported outcomes at week 52. Baseline is defined as the last non-missing assessment recorded on or prior to the date of first study drug injection at week 0. Data shown as mean (SD) at baseline, and mBOCF (SD) at week 52. ASAS = Assessment of Spondyloarthritis International Society; BASDAI = Bath Ankylosing Spondylitis Disease Activity Index; BASFI = Bath Ankylosing Spondylitis Functional Index; bDMARD = biologic disease-modifying anti-rheumatic drugs; IXE = ixekizumab; JSEQ = Jenkins Sleep Evaluation Questionnaire; mBOCF = modified baseline observation carried forward; N = number of patients in the analysis population; n = number of patients in each treatment subgroup; NRS = numeric rating scale; PtGA = patient global disease activity; Q = question; Q2W = every 2 weeks; Q4W = every 4 weeks; SD = standard deviation; TNFi = tumor necrosis factor inhibitor. **Figure S1.** Association between ASAS response and improvements in ASAS PROs for ixekizumab Q2W-treated patients after 52 weeks. Q2W ixekizumab-treated patients. § p<0.001, achieved ASAS20 but not ASAS40 vs. ASAS20 not achieved; * p<0.0001, ASAS40 achieved vs. ASAS20 not achieved; † p<0.0001, ASAS40 achieved vs. achieved ASAS20 but not ASAS40. Results were compared using ANCOVA. Values are LSM improvements from baseline (SE). mBOCF was used for imputation of missing data. Fold difference = (ASAS40 responder/ASAS20 non-responder) -1, or (ASAS40 responder/ASAS20 but not ASAS40 responder) -1. ANCOVA = analysis of covariance; ASAS = Assessment of Spondyloarthritis International Society; BASDAI = Bath Ankylosing Spondylitis Disease Activity Index; BASFI = Bath Ankylosing Spondylitis Functional Index; bDMARD = biologic disease-modifying antirheumatic drugs; LSM = least squares mean; mBOCF = modified baseline observation carried forward; Nx = number of observations; PROs = patient-reported outcomes; PtGA = patient global disease activity; Q = question; Q2W = every 2 weeks; SE: standard error; TNFi = tumor necrosis factor inhibitor. **Figure S2.** Association between ASAS response and improvements in ASAS PROs for ixekizumab Q4W-treated patients after 52 weeks. Q4W ixekizumab-treated patients. § p<0.001, achieved ASAS20 but not ASAS40 vs. ASAS20 not achieved; * p<0.0001, ASAS40 achieved vs. ASAS20 not achieved; † p<0.0001, ASAS40 achieved vs. achieved ASAS20 but not ASAS40. Results were compared using ANCOVA. Values are LSM improvements from baseline (SE). mBOCF was used for imputation of missing data. Fold difference = (ASAS40 responder/ASAS20 non-responder) -1, or (ASAS40 responder/ASAS20 but not ASAS40 responder) -1. ANCOVA = analysis of covariance; ASAS = Assessment of Spondyloarthritis International Society; BASDAI = Bath Ankylosing Spondylitis Disease Activity Index; BASFI = Bath Ankylosing Spondylitis Functional Index; bDMARD = biologic disease-modifying antirheumatic drugs; LSM = least squares mean; mBOCF = modified baseline observation carried forward; Nx = number of observations; PROs = patient reported outcomes; PtGA = patient global disease activity; Q = question; Q4W = every 4 weeks; SE = standard error; TNFi = tumor necrosis factor inhibitor. **Figure S3.** Association between ASAS response and improvements in other PROs for ixekizumab Q2W-treated patients after 52 weeks.Q2W ixekizumab-treated patients. § p<0.001, achieved ASAS20 but not ASAS40 vs. ASAS20 not achieved; * p<0.0001, ASAS40 achieved vs. ASAS20 not achieved; † p<0.0001, ASAS40 achieved vs. achieved ASAS20 but not ASAS40. Results were compared using ANCOVA. Values are LSM improvements from baseline (SE). mBOCF was used for imputation of missing data. Fold difference = (ASAS40 responder/ASAS20 non-responder) -1, or (ASAS40 responder/ASAS20 but not ASAS40 responder) -1. ANCOVA = analysis of covariance; ASAS = Assessment of Spondyloarthritis International Society; BASDAI = Bath Ankylosing Spondylitis Disease Activity Index; bDMARD = biologic disease-modifying antirheumatic drugs; JSEQ = Jenkins Sleep Evaluation Questionnaire; LSM = least squares mean; mBOCF: modified baseline observation carried forward; NRS = numeric rating scale; Nx = number of observations; PROs = patient reported outcomes; Q = question; Q2W = every 2 weeks; SE = standard error; TNFi = tumor necrosis factor inhibitor. **Figure S4.** Association between ASAS response and improvements in other PROs for ixekizumab Q4W-treated patients after 52 weeks.Q4W ixekizumab-treated patients. § p<0.001, achieved ASAS20 but not ASAS40 vs. ASAS20 not achieved; * p<0.0001, ASAS40 achieved vs. ASAS20 not achieved; † p<0.0001, ASAS40 achieved vs. achieved ASAS20 but not ASAS40. Results were compared using ANCOVA. Values are LSM improvements from baseline (SE). mBOCF was used for imputation of missing data. Fold difference = ASAS40 responder/ASAS20 non-responder) -1, or (ASAS40 responder/ASAS20 but not ASAS40 responder) -1. ANCOVA = analysis of covariance; ASAS = Assessment of Spondyloarthritis International Society; BASDAI = Bath Ankylosing Spondylitis Disease Activity Index; bDMARD = biologic disease-modifying antirheumatic drugs; JSEQ = Jenkins Sleep Evaluation Questionnaire; LSM = least squares mean; mBOCF = modified baseline observation carried forward; NRS = numeric rating scale; Nx = number of observations; PROs = patient-reported outcomes; Q = question; Q4W = every 4 weeks; SE = standard error; TNFi = tumor necrosis factor inhibitor.

## Data Availability

Data on all individual participant data collected during the trials, with the exception of pharmacokinetic or genetic data, are available upon request. Access is granted after an independent review committee’s approval and after receiving a signed data sharing agreement. Please contact the corresponding author for access to the datasets generated and/or analyzed during the current study.

## Abbreviations

ANCOVA: Analysis of covariance
AS: Ankylosing spondylitis
ASAS: Assessment of Spondyloarthritis International Society
BASDAI: Bath Ankylosing Spondylitis Disease Activity Index
BASFI: Bath Ankylosing Spondylitis Functional Index
bDMARD: Biologic disease-modifying antirheumatic drug
JSEQ: Jenkins Sleep Evaluation Questionnaire
mBOCF: Modified baseline observation carried forward
NRS: Numeric rating scale
PRO: Patient-reported outcome
PtGA: Patient global disease activity
Q2W: Every 2 weeks
Q4W: Every 4 weeks
r-axSpA: Radiographic axial spondyloarthritis
TNFi: Tumor necrosis factor inhibitor
