# Supplementary material for: Ixekizumab improves spinal pain, function, fatigue, stiffness, and sleep in radiographic axial Spondyloarthritis: COAST-V/W 52-week results
Source: BMC Rheumatol. 2021 Sep 20;5:35. doi: 10.1186/s41927-021-00205-3 (PMC8451150; doi:10.1186/s41927-021-00205-3)
Supplement: Supplementary file 1 — Additional file 1: Table S1. Changes from baseline in patient reported outcomes at week 52. Baseline is defined as the last non-missing assessment recorded on or prior to the date of first study drug injection at week 0. Data shown as mean (SD) at baseline, and mBOCF (SD) at week 52. ASAS = Assessment of Spondyloarthritis International Society; BASDAI = Bath Ankylosing Spondylitis Disease Activity Index; BASFI = Bath Ankylosing Spondylitis Functional Index; bDMARD = biologic disease-modifying anti-rheumatic drugs; IXE = ixekizumab; JSEQ = Jenkins Sleep Evaluation Questionnaire; mBOCF = modified baseline observation carried forward; N = number of patients in the analysis population; n = number of patients in each treatment subgroup; NRS = numeric rating scale; PtGA = patient global disease activity; Q = question; Q2W = every 2 weeks; Q4W = every 4 weeks; SD = standard deviation; TNFi = tumor necrosis factor inhibitor. Figure S1. Association between ASAS response and improvements in ASAS PROs for ixekizumab Q2W-treated patients after 52 weeks. Q2W ixekizumab-treated patients. § p<0.001, achieved ASAS20 but not ASAS40 vs. ASAS20 not achieved; * p<0.0001, ASAS40 achieved vs. ASAS20 not achieved; † p<0.0001, ASAS40 achieved vs. achieved ASAS20 but not ASAS40. Results were compared using ANCOVA. Values are LSM improvements from baseline (SE). mBOCF was used for imputation of missing data. Fold difference = (ASAS40 responder/ASAS20 non-responder) -1, or (ASAS40 responder/ASAS20 but not ASAS40 responder) -1. ANCOVA = analysis of covariance; ASAS = Assessment of Spondyloarthritis International Society; BASDAI = Bath Ankylosing Spondylitis Disease Activity Index; BASFI = Bath Ankylosing Spondylitis Functional Index; bDMARD = biologic disease-modifying antirheumatic drugs; LSM = least squares mean; mBOCF = modified baseline observation carried forward; Nx = number of observations; PROs = patient-reported outcomes; PtGA = patient global disease activity; Q = question; Q2W = eve [file 41927_2021_205_MOESM1_ESM.docx]

**Supplementary material**

**Supplementary Table S1. Changes from baseline in patient reported outcomes at week 52.**

|  |  | COAST-V: bDMARD-naïve  (N=329) | | COAST-W: TNFi-experienced  (N=281) | |
| --- | --- | --- | --- | --- | --- |
|  | **Week** | **IXE Q4W**  **(n=78)** | **IXE QW2**  **(n=79)** | **IXE Q4W**  **(n=98)** | **IXE Q2W**  **(n=90)** |
| ASAS Response Domains |  |  |  |  |  |
| PtGA | 0 | 6.9 (1.5) | 7.1 (1.6) | 7.9 (1.7) | 7.8 (1.7) |
|  | 52 | -3.2 (2.8) | -3.4 (2.5) | -3.0 (2.8) | -2.6 (2.8) |
| Spinal Pain | 0 | 7.2 (1.3) | 7.2 (1.5) | 7.9 (1.5) | 7.8 (1.6) |
|  | 52 | -3.8 (2.7) | -3.7 (2.5) | -3.1 (2.7) | -2.8 (2.7) |
| Stiffness  (BASDAI Q5 & Q6) | 0 | 6.6 (1.6) | 6.8 (1.9) | 7.1 (1.6) | 7.4 (1.7) |
|  | 52 | -3.5 (2.7) | -3.5 (2.3) | -2.9 (2.5) | -3.0 (2.6) |
| BASFI | 0 | 6.1 (1.8) | 6.3 (2.1) | 7.2 (1.8) | 7.4 (1.4) |
|  | 52 | -2.9 (2.4) | -2.9 (2.4) | -2.4 (2.5) | -2.3 (2.3) |
| Other Outcomes |  |  |  |  |  |
| Spinal Pain at Night | 0 | 7.1 (1.4) | 7.1 (1.6) | 7.9 (1.4) | 7.7 (1.6) |
|  | 52 | -4.0 (2.7) | -3.7 (2.6) | -3.4 (2.7) | -2.9 (2.9) |
| Fatigue  (BASDAI Q1) | 0 | 7.2 (1.5) | 7.2 (1.7) | 7.6 (1.4) | 7.7 (1.5) |
|  | 52 | -3.2 (2.8) | -3.2 (2.5) | -2.5 (2.5) | -2.4 (2.5) |
| Fatigue NRS | 0 | 6.7 (1.7) | 6.7 (1.7) | 7.5 (1.7) | 7.2 (2.0) |
|  | 52 | -2.8 (2.7) | -2.6 (2.6) | -2.8 (2.5) | -2.0 (2.7) |
| Sleep (JSEQ) | 0 | 7.1 (5.3) | 8.7 (5.1) | 9.9 (5.8) | 10.4 (5.4) |
|  | 52 | -2.2 (4.3) | -3.7 (4.6) | -3.8 (5.9) | -2.7 (5.5) |
| BASDAI | 0 | 6.8 (1.3) | 6.7 (1.6) | 7.4 (1.3) | 7.4 (1.2) |
|  | 52 | -3.4 (2.4) | -3.2 (2.3) | -2.8 (2.3) | -2.5 (2.3) |

Baseline is defined as the last non-missing assessment recorded on or prior to the date of first study drug injection at week 0. Data shown as mean (SD) at baseline, and mBOCF (SD) at week 52.

ASAS = Assessment of Spondyloarthritis International Society; BASDAI = Bath Ankylosing Spondylitis Disease Activity Index; BASFI = Bath Ankylosing Spondylitis Functional Index; bDMARD = biologic disease-modifying anti-rheumatic drugs; IXE = ixekizumab; JSEQ = Jenkins Sleep Evaluation Questionnaire; mBOCF = modified baseline observation carried forward; N = number of patients in the analysis population; n = number of patients in each treatment subgroup; NRS = numeric rating scale; PtGA = patient global disease activity; Q = question; Q2W = every 2 weeks; Q4W = every 4 weeks; SD = standard deviation; TNFi = tumor necrosis factor inhibitor.

**Supplementary Figure S1. Association between ASAS response and improvements in ASAS PROs for ixekizumab Q2W-treated patients after 52 weeks.**


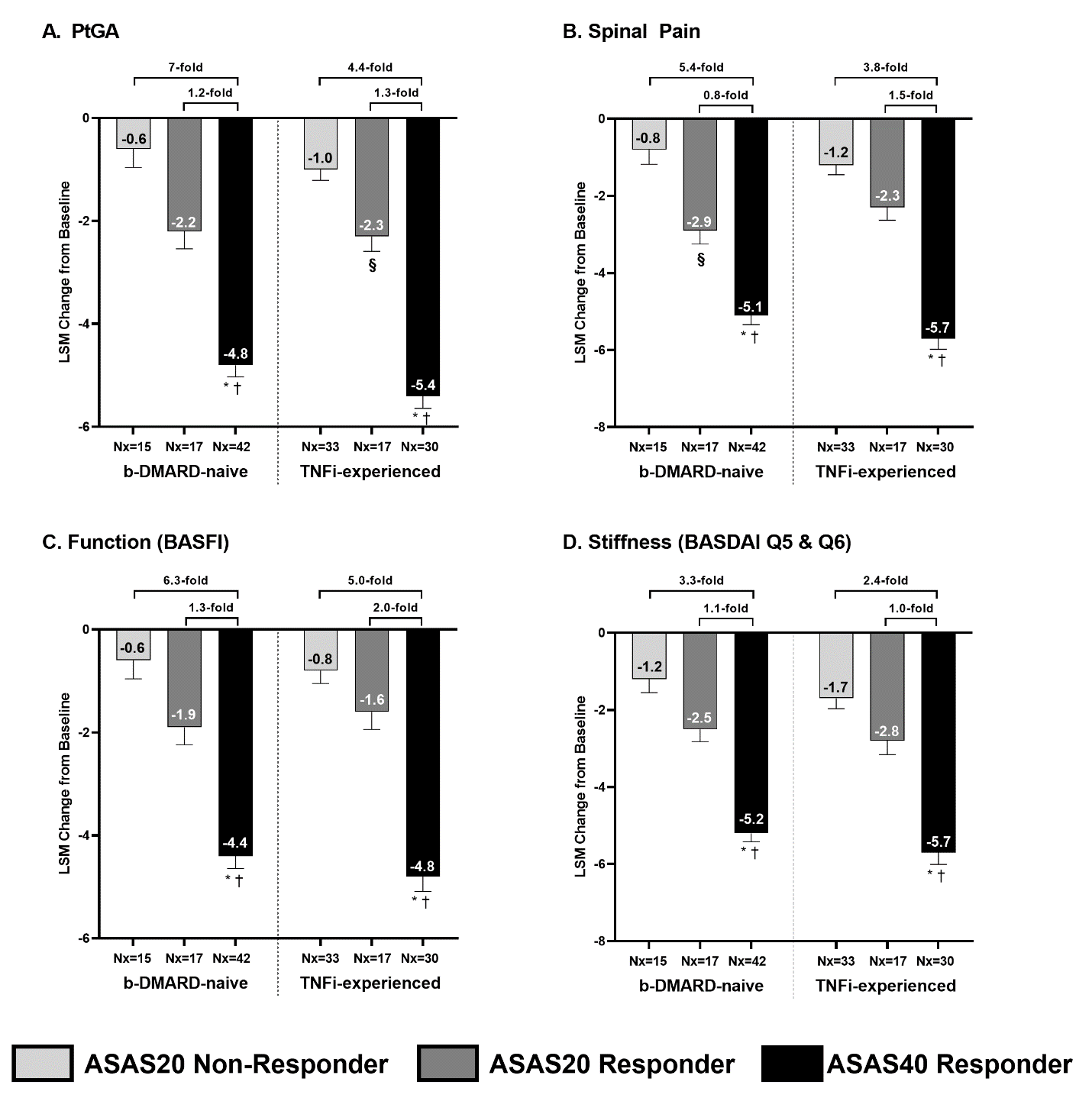


Q2W ixekizumab-treated patients. § p<0.001, achieved ASAS20 but not ASAS40 vs. ASAS20 not achieved; * p<0.0001, ASAS40 achieved vs. ASAS20 not achieved; † p<0.0001, ASAS40 achieved vs. achieved ASAS20 but not ASAS40. Results were compared using ANCOVA. Values are LSM improvements from baseline (SE). mBOCF was used for imputation of missing data. Fold difference = (ASAS40 responder/ASAS20 non-responder) -1, or (ASAS40 responder/ASAS20 but not ASAS40 responder) -1.

ANCOVA = analysis of covariance; ASAS = Assessment of Spondyloarthritis International Society; BASDAI = Bath Ankylosing Spondylitis Disease Activity Index; BASFI = Bath Ankylosing Spondylitis Functional Index; bDMARD = biologic disease-modifying antirheumatic drugs; LSM = least squares mean; mBOCF = modified baseline observation carried forward; Nx = number of observations; PROs = patient-reported outcomes; PtGA = patient global disease activity; Q = question; Q2W = every 2 weeks; SE: standard error; TNFi = tumor necrosis factor inhibitor.

**Supplementary Figure S2. Association between ASAS response and improvements in ASAS PROs for ixekizumab Q4W-treated patients after 52 weeks.**


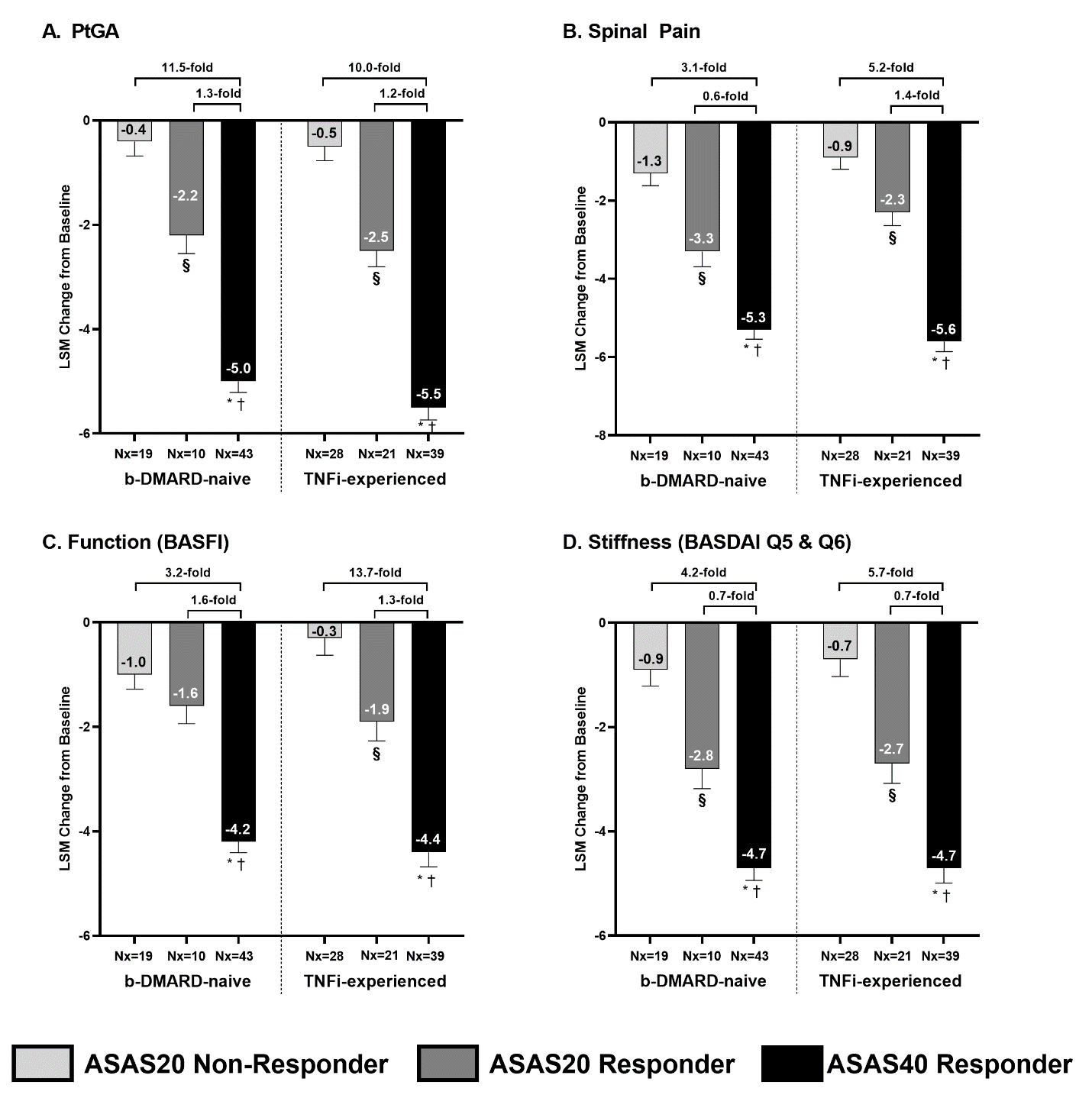


Q4W ixekizumab-treated patients. § p<0.001, achieved ASAS20 but not ASAS40 vs. ASAS20 not achieved; * p<0.0001, ASAS40 achieved vs. ASAS20 not achieved; † p<0.0001, ASAS40 achieved vs. achieved ASAS20 but not ASAS40. Results were compared using ANCOVA. Values are LSM improvements from baseline (SE). mBOCF was used for imputation of missing data. Fold difference = (ASAS40 responder/ASAS20 non-responder) -1, or (ASAS40 responder/ASAS20 but not ASAS40 responder) -1.

ANCOVA = analysis of covariance; ASAS = Assessment of Spondyloarthritis International Society; BASDAI = Bath Ankylosing Spondylitis Disease Activity Index; BASFI = Bath Ankylosing Spondylitis Functional Index; bDMARD = biologic disease-modifying antirheumatic drugs; LSM = least squares mean; mBOCF = modified baseline observation carried forward; Nx = number of observations; PROs = patient reported outcomes; PtGA = patient global disease activity; Q = question; Q4W = every 4 weeks; SE = standard error; TNFi = tumor necrosis factor inhibitor.

**Supplementary Figure S3. Association between ASAS response and improvements in other PROs for ixekizumab Q2W-treated patients after 52 weeks.**


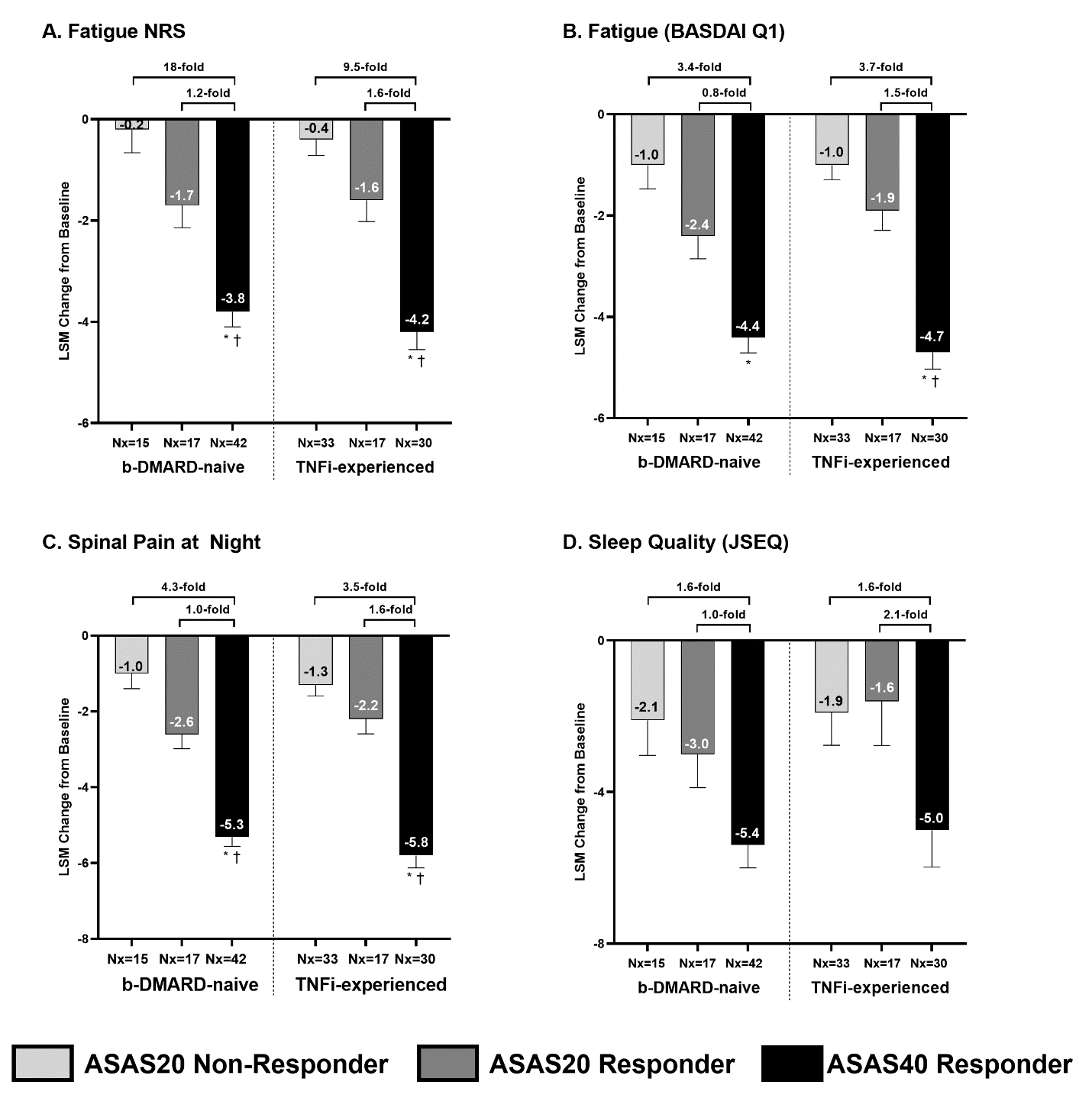


Q2W ixekizumab-treated patients. § p<0.001, achieved ASAS20 but not ASAS40 vs. ASAS20 not achieved; * p<0.0001, ASAS40 achieved vs. ASAS20 not achieved; † p<0.0001, ASAS40 achieved vs. achieved ASAS20 but not ASAS40. Results were compared using ANCOVA. Values are LSM improvements from baseline (SE). mBOCF was used for imputation of missing data. Fold difference = (ASAS40 responder/ASAS20 non-responder) -1, or (ASAS40 responder/ASAS20 but not ASAS40 responder) -1.

ANCOVA = analysis of covariance; ASAS = Assessment of Spondyloarthritis International Society; BASDAI = Bath Ankylosing Spondylitis Disease Activity Index; bDMARD = biologic disease-modifying antirheumatic drugs; JSEQ = Jenkins Sleep Evaluation Questionnaire; LSM = least squares mean; mBOCF: modified baseline observation carried forward; NRS = numeric rating scale; Nx = number of observations; PROs = patient reported outcomes; Q = question; Q2W = every 2 weeks; SE = standard error; TNFi = tumor necrosis factor inhibitor.

**Supplementary Figure S4. Association between ASAS response and improvements in other PROs for ixekizumab Q4W-treated patients after 52 weeks.**


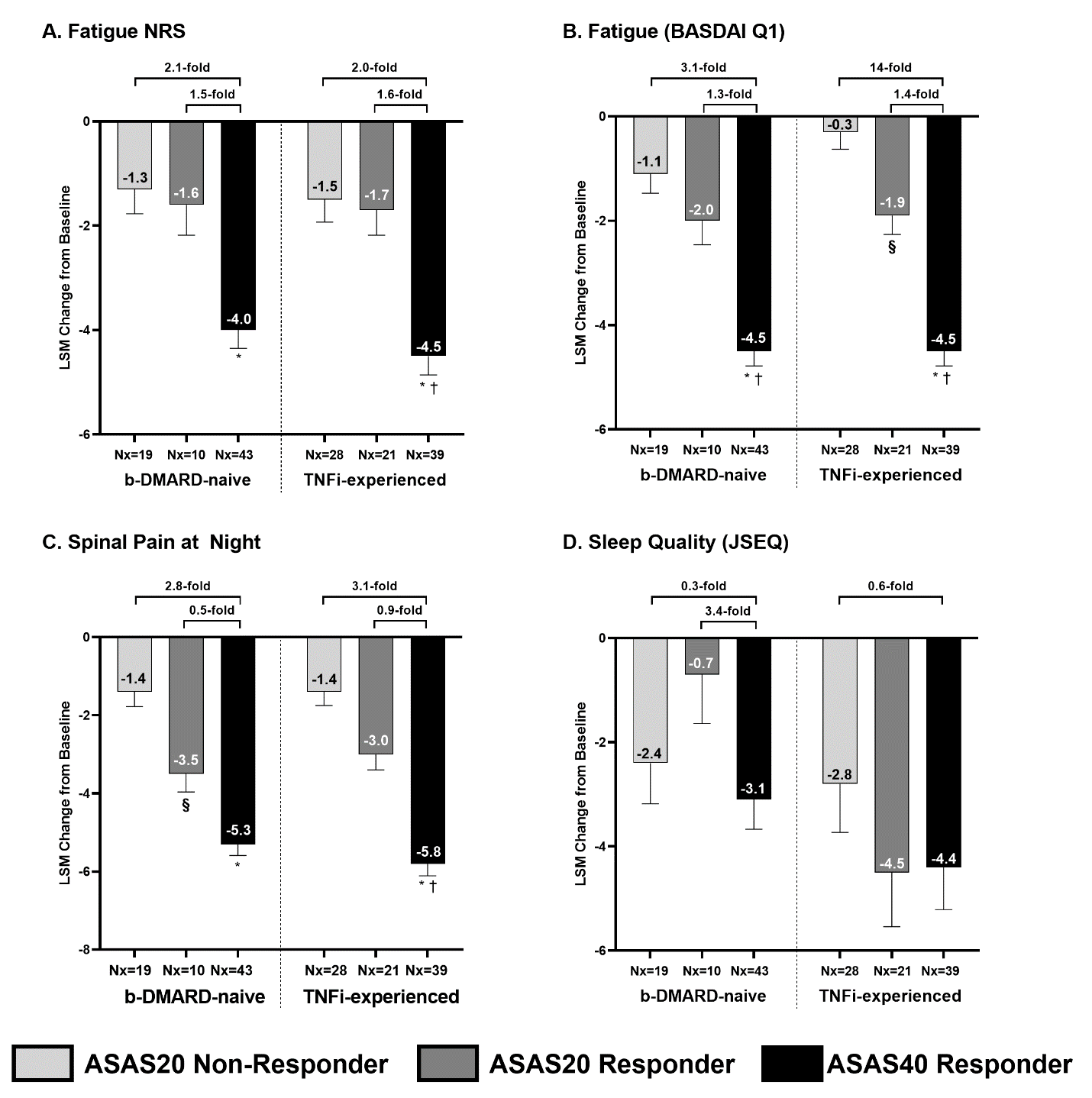


Q4W ixekizumab-treated patients. § p<0.001, achieved ASAS20 but not ASAS40 vs. ASAS20 not achieved; * p<0.0001, ASAS40 achieved vs. ASAS20 not achieved; † p<0.0001, ASAS40 achieved vs. achieved ASAS20 but not ASAS40. Results were compared using ANCOVA. Values are LSM improvements from baseline (SE). mBOCF was used for imputation of missing data. Fold difference = ASAS40 responder/ASAS20 non-responder) -1, or (ASAS40 responder/ASAS20 but not ASAS40 responder) -1.

ANCOVA = analysis of covariance; ASAS = Assessment of Spondyloarthritis International Society; BASDAI = Bath Ankylosing Spondylitis Disease Activity Index; bDMARD = biologic disease-modifying antirheumatic drugs; JSEQ = Jenkins Sleep Evaluation Questionnaire; LSM = least squares mean; mBOCF = modified baseline observation carried forward; NRS = numeric rating scale; Nx = number of observations; PROs = patient-reported outcomes; Q = question; Q4W = every 4 weeks; SE = standard error; TNFi = tumor necrosis factor inhibitor.
